# Differential Protein Distribution between the Nucleus and Mitochondria: Implications in Aging

**DOI:** 10.3389/fgene.2016.00162

**Published:** 2016-09-16

**Authors:** Eirini Lionaki, Ilias Gkikas, Nektarios Tavernarakis

**Affiliations:** ^1^Institute of Molecular Biology and Biotechnology, Foundation for Research and Technology-HellasHeraklion, Greece; ^2^Department of Basic Sciences, Faculty of Medicine, University of CreteHeraklion, Greece

**Keywords:** aging, anterograde signaling, electron transport chain, mitochondrial targeting, nuclear localization, organellar protein distribution, retrograde signaling, cell death

## Abstract

The coordination of nuclear and mitochondrial genomes plays a pivotal role in maintenance of mitochondrial biogenesis and functionality during stress and aging. Environmental and cellular inputs signal to nucleus and/or mitochondria to trigger interorganellar compensatory responses. Loss of this tightly orchestrated coordination results in loss of cellular homeostasis and underlies various pathologies and age-related diseases. Several signaling cascades that govern interorganellar communication have been revealed up to now, and have been classified as part of the anterograde (nucleus to mitochondria) or retrograde (mitochondrial to nucleus) response. Many of these molecular pathways rely on the dual distribution of nuclear or mitochondrial components under basal or stress conditions. These dually localized components usually engage in specific tasks in their primary organelle of function, whilst upon cellular stimuli, they appear in the other organelle where they engage in the same or a different task, triggering a compensatory stress response. In this review, we focus on protein factors distributed between the nucleus and mitochondria and activated to exert their functions upon basal or stress conditions. We further discuss implications of bi-organellar targeting in the context of aging.

## Introduction

Nucleus and mitochondria are the two main genome-bearing organelles of the eukaryotic cell with central and orchestrating roles in cellular and organismal physiology. Mitochondria lay at the core of cellular metabolism with critical involvement in many enzymatic and signaling pathways affecting energy production, cell proliferation, differentiation and survival. Besides the fact that mitochondria contain their own genome, mitochondrial DNA (mtDNA) encodes only for ∼1% of the total mitochondrial proteome. The rest 99% is encoded by nuclear genes, translated in the cytoplasm and then translocated in the sub-mitochondrial compartment to which they are destined. A significant proportion of mitochondrial proteins are translated by ribosomes that reside on the outer mitochondrial membrane and are imported co-translationally (Lesnik et al., 2014). The expression of nuclear and mitochondrial DNA needs to be tuned for proper and timely assembly of mitochondrial protein complexes. This interorganellar coordination becomes even more critical under stress conditions, when both the nucleus and the mitochondrion need to adjust their functions accordingly, so that the cell can cope with the imposed stress.

Mitonuclear communication is governed by key factors and signaling cascades that have been identified in different species and are classified as anterograde or retrograde signaling. Anterograde signaling includes the most traditional perspective of the nucleus as regulatory factor coordinating the function of subcellular organelles. The nucleus integrates cellular and environmental signals to adjust its function and the function of other organelles. Usually, this involves the function of transcription factors regulating gene expression. Several transcription factors have been shown to regulate distinct classes of mitochondrial genes. In mammalian cells, these are nuclear respiratory factor 1 (NRF1), activating genes involved in respiration, heme biosynthesis, mitochondrial DNA replication and transcription (Scarpulla et al., 2012), Nuclear Factor Erythroid 2-Like 2 (NFE2L2 or NRF2) or GA-Binding Protein (GABD) activating genes regulating cytochrome c oxidase assembly, ATP synthesis and inner mitochondrial membrane potential (Dinkova-Kostova and Abramov, 2015), and members of the nuclear receptor superfamily like peroxisome proliferator-activated receptors (PPARs) and estrogen-related receptors (ERRs), with key roles in the regulation of fatty acid beta-oxidation and oxidative phosphorylation, respectively (Fan and Evans, 2015). The function of all these classes of transcription factors on mitochondrial metabolism is fine-tuned by specific transcriptional co-activators. PGC-1a is a transcriptional co-activator of NRF1, NFE2L2, PPARs, and ERRs, among others, and is known to enhance virtually all aspects of mitochondrial biology, including respiratory capacity, fatty-acid oxidation, and mitochondrial content. Many environmental and cellular signals, like cold exposure, nutrient deprivation or exercise, converge on PGC-1a to modulate in concert, mitochondrial metabolism. Moreover, molecular factors affecting expression on PGC1a in a tissue-specific manner allow tissues with divergent metabolic needs to regulate their energetic status accordingly [for an extended review of anterograde signaling see (Scarpulla et al., 2012)]. Homologs of PGC-1a have not been found in *Drosophila melanogaster* or *Caenorhabditis elegans*. However, in *D. melanogaster* Erect Wing (ERW), the homolog of NRF1, was recently shown to activate mitochondrial fusion through upregulation of OPA1-like (Rai et al., 2014), while in *C. elegans* we have recently identified a NFE2L2 homolog, SKN-1, to be required for gene transcription of several mitochondrial proteins (Palikaras et al., 2015).

Reversely, mitochondria can sense stress signals and convey them to the nucleus to initiate adaptive responses. This type of signaling from a peripheral organelle to the nucleus is known as retrograde response. Mitochondrial retrograde response is amongst the most well studied retrograde signaling pathways. It was firstly described in *Saccharomyces cerevisiae* where the retrograde response genes (RTGs) mediate mitochondrial signals (mainly reactive oxygen species) to the nucleus to trigger an adaptive transcriptional response leading to enhanced replicative lifespan (Liao and Butow, 1993; Srinivasan et al., 2010). Representative metabolic adaptations induced by retrograde signaling are the glyoxylate cycle and fatty acid beta oxidation (the latter performed in peroxisomes in yeast). In mammals there are no direct homologs of RTGs, however, the retrograde response upon mitochondrial dysfunction is mediated by nuclear translocation of transcription factors like NF-kB and cAMP response element-binding protein (CREB; Arnould et al., 2002) among others. These transcription factors respond to oxidative stress, intracellular calcium levels, inhibition of electron transport chain (ETC), or mitochondrial proteotoxic stress. For a recent review of mitochondrial retrograde signaling see (da Cunha et al., 2015; Quiros et al., 2016).

Apart from these complex signaling cascades that mediate the communication of nucleus to mitochondrial and vice versa a more direct way of interorganellar coordination has emerged, which relies on the redistribution of nuclear or mitochondrial proteins between the two compartments. Nuclear proteins with mitochondrial distribution are mainly transcription factors that translocate to mitochondria upon a stress stimulus or reside in mitochondria under steady state conditions and are activated upon stress. Mitochondrial proteins with nuclear distribution are transcription factors with roles both in mitochondrial and nuclear DNA, biosynthetic enzymes or pro-apoptotic factors. To a great extent, this dual localization of proteins can be achieved through the presence of two (or more) targeting signals within the amino acid sequence which are revealed under specific conditions. It is conceivable that this level of interorganellar communication and coordination is very important as it allows for direct and finely tuned responses of both organelles leading to enhanced, precise and acute stress adaptation. In this review we will focus on nuclear and mitochondrial proteins with dual distribution between nucleus and mitochondria and their roles in the heterologous compartment. Finally, we will discuss the reported implications of this bi-organellar coordination in the process of aging.

## Nuclear Proteins Targeting Mitochondria

**FIGURE 1:**
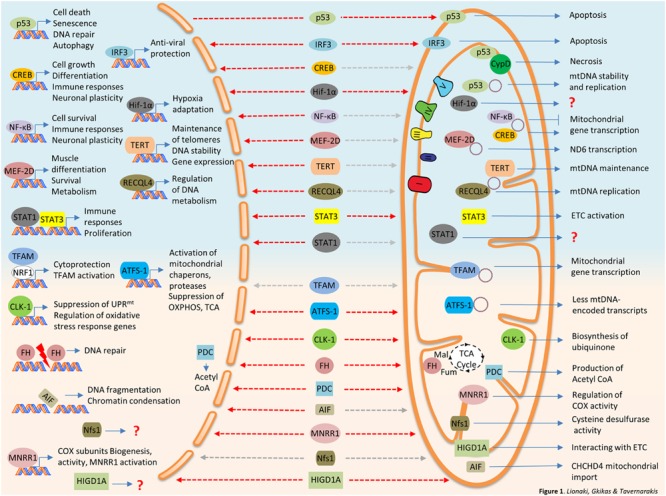
**Bi-organellar protein trafficking between nucleus and mitochondria.** Nuclear proteins traveling to mitochondria are summarized at the top panel of the figure (light blue background). Mitochondrial protein traveling to the nucleus are described at the bottom panel of the figure (pink background). Destinations are marked with dashed arrows, red for stress-induced trafficking/activation and gray for steady state or undefined stimulus. AIF, Apoptosis-Inducing Factor; ATFS-1, activating transcription factor 1; CLK-1, Clock 1; CREB, cAMP response element-binding protein; CypD, Cyclophilin D; FH, fumarase; IRF3, interferon regulated factor 3; HIGDA1, hypoxia-inducible gene domain 1A; HIF-1α, Hypoxia-Inducible Factor 1α; MEF-2D, Myocyte Enhancer Factor-2D; MNRR1, mitochondrial nuclear retrograde regulator 1; ND6, NADH dehydrogenase 6; NF-κB, nuclear factor kappa-light-chain-enhancer of activated B cells; Nfs1, NFS1 Cysteine Desulfurase; PDC, Pyruvate dehydrogenase complex; RECQL4, RecQ like helicase 4; STAT1, signal transducer and activator of transcription 1; STAT3, signal transducer and activator of transcription 3; TERT, Telomerase reverse transcriptase.

In the following section we will focus on predominantly nuclear proteins, mainly transcription factors, found to reside in mitochondria, either under steady state conditions or upon cellular or environmental signals (**Table 1**; **Figure 1**). The dual distribution of these transcription factors have been mainly studied in mammalian cells thus we will focus on mammalian studies.

**Table 1:** Nuclear proteins with defined roles in mitochondria.

Protein	Species	Mitochondrial function	Stimulus driving mitochondrial localization	Reference
NF-κB	Rat, mouse	Suppression of mitochondrial genes	Steady-state conditions	Johnson et al., 2011; Johnson and Perkins, 2012
CREB	Rat, mouse	Activation of mitochondrial genes	Steady-state conditions	Lee et al., 2005; Ryu et al., 2005
MEF-2D	Mouse	Transcriptional Activation of ND6	Steady-state conditions	She et al., 2011
TERT	Human, mouse, Rat	Reverse Transcription of mitochondrial tRNAs, Protection from mtDNA damage	Oxidative stress, steady state conditions	Haendeler et al., 2009; Sharma et al., 2012; Saretzki, 2014
RECQL4	Human, mouse	Restores mtDNA replication, Ensures mtDNA integrity	Steady-state conditions	De et al., 2012; Gupta et al., 2014; Wang et al., 2014; Lu et al., 2016
STAT3	Mouse, human	Modulation ETC through direct binding to respiratory complexes	Steady-state conditions Activated Ras	Gough et al., 2009; Wegrzyn et al., 2009
P53	Rat, mouse, human	Modulation of mitochondrial permeability, apoptosis and necrosis, Promotion of mtDNA accuracy and stability.	Pro-apoptotic signals, oxidative stress, hypoxia, ultraviolet irradiation	Jiang et al., 2006; Marchenko et al., 2007; Bakhanashvili et al., 2008; Vaseva et al., 2012
IRF3	Human	Apoptosis induction by mitochondrial recruitment of BAX	RNA virus infection	Chattopadhyay et al., 2010
STAT1	Mouse, Rat	ND	Steady-state conditions	Boengler et al., 2010
HIF-1α	Human	ND	Hypoxia	Briston et al., 2011

*ND, Not defined.*

### Nuclear Proteins Modulating Mitochondrial DNA Expression

#### NF-κB

NF-κB (nuclear factor kappa-light-chain-enhancer of activated B cells) is a dimeric transcription factor. The NF-κB family consists of five members; p65 (RelA), p105/p50 (NF-κB1), p100/p52 (NF-κB2), c-Rel and RelB. Normally NF-κB resides in the cytoplasm in complex with its inhibitor IκB (Inhibitor of κB). Upon stimulation, IkB is being phosphorylated and subsequently degraded, unmasking the nuclear targeting signal of NF-κB which is now free to translocate to the nucleus. Stimulation comes by various stress signals like inflammatory cytokines, low oxygen tension, bacterial lipopolysaccharide (LPS), elevated cytoplasmic calcium levels or DNA damage (Perkins, 2007). Upon stimulation, nuclear NF-κB activates transcription of genes that promote cell survival. Apart from its well-established role in the nucleus, NF-κB (p50 and RelA/p65 subunits), together with his inhibitor IκBα was shown to target mammalian mitochondria. Upon TNFα stimulation the mitochondrial pool of IκBα (member of IκB protein family) is being degraded and NF-κB negatively regulates mitochondrial gene expression in proliferating rat liver cells (Cogswell et al., 2003). Recently, it was shown that RelA (p65) binds to mitochondrial DNA and directly regulates mitochondrial gene transcription and thus mitochondrial respiration capacity and ATP production (Johnson et al., 2011). RelA mitochondrial translocation relays on its binding to mortalin (mtHSP70), the dedicated chaperone that pulls preproteins through the Translocase of the Inner Membrane (TIM23). Interestingly, p53 negatively regulates mitochondrial translocation of RelA. Thus, upon p53 loss, common feature of tumorigenesis, RelA translocates to mitochondria and shuts down oxidative metabolism probably contributing to cancer cell malignancy (Johnson and Perkins, 2012).

#### cAMP Response Element-Binding Protein (CREB)

The cAMP response element-binding protein (CREB) is a ubiquitous transcription factor that regulates cellular growth, proliferation and differentiation with critical roles in neuronal plasticity and long term memory formation in the brain, as well as with specific functions in immune responses (Wen et al., 2010; Sakamoto et al., 2011). Defects on CREB signaling have been linked with cognitive impairment similar to that observed in normal aging and neurodegeneration (Mantamadiotis et al., 2002; Pugazhenthi et al., 2011). CREB is activated through phosphorylation by several kinases like AMP-dependent protein kinase A (PKA), protein kinase C (PKC), calmodulin kinases, responding to messengers like cAMP and/or intracellular calcium fluxes. Interestingly, CREB has been found within the mitochondrial matrix of neurons of adult rat cortex (Lee et al., 2005). It is found in mitochondria under steady state condition in cultured neurons. It is not clear whether mitochondrial accumulation of CREB is regulated by specific cellular signals or whether it happens constitutively. CREB lacks a classical mitochondrial targeting signal (MTS), nevertheless, it enters the mitochondrial matrix in Δψ-dependent manner, through the Translocase of the Outer Membrane, TOM complex (De Rasmo et al., 2009). Mitochondrial CREB was shown to bind to CRE-like sites within the D-loop of mtDNA triggering the transcription of several mitochondrial genes (Lee et al., 2005; Ryu et al., 2005). It has been proposed that mitochondrial CREB is activated by mitochondrial PKA in the matrix, in response to Deferoxamine (DFO), an iron chelator and antioxidant which protects against oxidative stress induced neuronal apoptosis (Ryu et al., 2005). Therefore, it is suggested that low ROS levels can activate mitochondrial CREB which, in turn, enhances mitochondrial gene expression, oxidative phosphorylation and ultimately promote neuronal survival. It has been shown that cAMP can be produced within the mitochondrial matrix by soluble adenyl cyclase (sAC) that resides locally (Valsecchi et al., 2013). sAC is regulated by calcium levels, ATP levels and bicarbonate (Chen et al., 2000; Litvin et al., 2003). Downstream effectors of cAMP, like PKA, are also found in the matrix. This raises the possibility that local changes of cAMP levels induced by fluctuations in calcium, ATP or bicarbonate levels could directly activate the mitochondrial pool of CREB leading to modulation of mitochondrial metabolism.

#### Myocyte Enhancer Factor-2D (MEF2D)

Myocyte Enhancer Factor-2D (MEF2D) belongs to the Myocyte enhancer factor 2 (MEF2) family of transcription factors with important roles in muscle differentiation, immune cell responses, glucose metabolism (in adipocytes), cellular development and survival (in neurons; Brusco and Haas, 2015). In neurons, MEF2D binds on nuclear DNA in order to modulate the synapses and on mtDNA to activate expression of NADH dehydrogenase 6 (ND6). ND6 is a mitochondrial gene critical for Complex I assembly and function, cellular respiration and ATP production (She et al., 2011). Specifically, a dominant negative mutant of MEF2D caused reduction of Complex I protein levels and activity without affecting complexes II–V. Moreover, it caused reduced ATP levels and increased H_2_O_2_ production. Overexpression of ND6 in MED2D-independent manner rescued complex I activity defect of MEF2D dominant negative mutant (MEF2Ddn). Mitochondrial dysfunction caused by two inhibitors of complex I, 1-methyl-4-phenylpyridinium [mitochondrial processing peptidase (MPP+)] and rotenone, decreased binding of MEF2D on ND6 gene and protein levels of mitochondrial MEF2D and ND6 protein, without affecting MEF2D nuclear pool (She et al., 2011). Collectively, these data suggest that direct regulation of Complex I activity by MED2D underlies its neuroprotective effects within mitochondria.

### Nuclear Proteins Involved in Mitochondrial DNA Maintenance

#### Telomerase Reverse Transcriptase (TERT)

Telomerase is a ribonucleoprotein with multiple functions. Primarily, it is responsible for telomere elongation (Cohen et al., 2007). It consists of the catalytic subunit, the enzyme telomerase reverse transcriptase (TERT) and the telomerase RNA component (TERC or TR) which together form the core functional unit of the telomerase complex. Proper telomere maintenance *in vivo* requires the holoenzyme of telomerase that comprises several interactors (Hukezalie and Wong, 2013). If telomerase is absent, telomeres shorten with each cycle of cell division reaching a critical threshold that induces a DNA damage response and cell cycle arrest leading to senescence (Herbig et al., 2004). A growing body of evidence suggests that besides its canonical functions, TERT has non-telomere-related functions in the nucleus, cytoplasm and mitochondria (Masutomi et al., 2005; Sarin et al., 2005; Choi et al., 2008; Lee et al., 2008; Maida et al., 2009; Chiodi and Mondello, 2012; Saretzki, 2014). These non-canonical functions include regulation of gene expression, mitochondrial function and interaction with intracellular signaling cascades such as NF-κB and WNT-β-catenin. Thus, TERT function affects cell survival and stress resistance, with important implications in inflammation and cancer invasiveness (Saretzki, 2014).

Telomerase reverse transcriptase affects mitochondrial function in distinct ways. Transcriptome analysis of proliferative and quiescent tissues from fourth generation *Tert^-^*^/-^ mice (G4), exhibiting a pronounced telomere dysfunction, showed decreased levels of PGC1a and PGC1β, and their critical targets, leading to reduced mitochondrial mass, reduced mtDNA content, oxygen consumption and energy production (Sahin et al., 2011). Moreover, decreased expression of ROS detoxifying enzymes was observed, leading to increased ROS and carbonylated proteins in both proliferative and non-proliferative tissues. The same results were obtained with *Terc*^-/-^ mice indicating that telomere dysfunction *per se* is triggering the observed mitochondrial abnormalities (Sahin et al., 2011).

Apart from the nuclear function of TERT affecting mitochondrial function, TERT is known to physically target the organelle. In higher eukaryotes (human, mouse, and rat) TERT contains a bipartite nuclear targeting signal, that regulates its shuttling in and out of the nucleus, and amitochondrial targeting sequence (MTS), that guides a fraction of endogenous TERT to the mitochondrial matrix. Mitochondrial TERT is imported in a membrane potential-dependent manner and it is localized in close proximity with the inner membrane (Santos et al., 2004; Sharma et al., 2012). Thus, TERT is found in both organelles under steady-state conditions in several cell types and also in non-dividing cells. However, oxidative stress induces hTERT nuclear export in a manner that depends on Src kinase and tyrosine 707 phosphorylation, followed by binding to Ran GTPase (Haendeler et al., 2003). Nuclear exported TERT accumulates to mitochondria and improves their function while protecting from acute and chronic oxidative stress by diminishing mitochondrial superoxide production and cellular ROS levels (Ahmed et al., 2008). Mitochondrial TERT co-fractionates with mtDNA and protects it from ethidium bromide-induced damage (Haendeler et al., 2009). A modified chromatin immune-precipitation assay with cells overexpressing hTERT suggested that it binds to several regions of mtDNA, including the 12S and 16S rRNAs, ND1, 2, 4, and 5, COX I and III and several mitochondrial tRNAs among others (Sharma et al., 2012). Conversely, hTERC is not present in mitochondria and is not required for the mitochondrial functions of hTERT. But what is the mysterious role of TERT in mitochondria? Latest findings suggest that TERT is able to use mitochondrial tRNAs to drive first strand synthesis *in vitro*, even in the absence of its specific RNA component TERC. Thus, TERT can function like other cellular reverse transcriptase within mitochondria using mitochondria tRNAs as template for reverse transcription. The function of mitochondrial TERT is critical both for the organelle and for the cellular physiology, as mutation of the targeting signal that excluded TERT from mitochondria but allowed it to enter and function properly in the nucleus, exhibited increased mtDNA damage and increased ROS production, while electron microscopy experiments revealed that more than 50% of *htert*^-/-^ mitochondria have an abnormal morphology. Cells expressing the nuclear form of TERT displayed increased autophagosomes containing mitochondria, suggesting elevated mitophagy rate, which is in line with the fact that TERT KO mice display reduced mitochondrial content (Sarin et al., 2005; Sharma et al., 2012). Interestingly, a hTERT mutant with defect in the nuclear export signal (NES), which disrupts the nucleus-cytoplasm shuttling of the protein, fails to immortalize cells while keeping its catalytic activity *in vitro* (Kovalenko et al., 2010). Moreover, cells expressing NES-defective hTERT display nuclear DNA damage and induce the DNA-damage response, pointing to the notion that non-nuclear roles of hTERT are also important for telomere maintenance.

#### RecQ Like Helicase 4 (RECQL4)

RecQL4 (RecQ Like Helicase 4) belongs to the human family of RECQ DNA helicases and its role in DNA metabolism has been well-documented. Upon laser-induced DNA double strand breaks (DSBs), nuclear RECQL4 is accumulated at the DNA break sites. RECQL4 deficient cells failed to repair DNA DSBs and were sensitive upon exposure to γ-irradiation, suggesting a role of RECQL4 in DNA repair process (Singh et al., 2010). In human cells, it was also shown that RECQL4 co-localize and co-immunoprecipitate with RAD51, an essential factor for homologous recombination and DNA repair (Petkovic et al., 2005). Alongside the DNA repair role, RECQL4 interacts with proteins of the mini-chromosome maintenance complex (MCM) which are implicated in the DNA replication. Mutants of human RECQL4 loss their ability to bind MCM10 and the efficiency of DNA replication is reduced (Im et al., 2015; Kliszczak et al., 2015; Lu et al., 2016). Mutations in *RECQL4* have been associated with three severe autosomal recessive diseases, the Rothmund–Thomson syndrome (RTS), RAPDILINO and Baller–Gerold syndrome (BGS) (Kitao et al., 1999; Dietschy et al., 2007). However, cancer is the most common cause of death for patient with *RECQL4* mutations (Croteau et al., 2014; Lu et al., 2016). RECQL4 is the only member of the human RECQ DNA helicases protein family that contains both nuclear and MTSs, allowing its shuttling between the nucleus and mitochondria, in cell-type specific manner (Burks et al., 2007; Croteau et al., 2012b). RECQL4 is localized to mitochondria in cell-cycle dependent manner, under normal conditions (Croteau et al., 2012a; De et al., 2012). Nuclear to mitochondria translocation of RECQL4 is negatively regulated by P23 activity (Wang et al., 2014). In the absence of DNA damage, RECQL4 physically interacts and masks the NTS of P53 preventing its nuclear localization (De et al., 2012). Binding of RECQL4 to P53 allows both proteins to co-localize in the mitochondrial nucleoids. Human cells lacking mtDNA failed to accumulate RECQL4 and P53 in the mitochondrial nucleoids. Mitochondrial RECQL4 and P53 are implicated in *de novo* replication of mtDNA. Cells of RTS patient, with a restored RECQL4 function, show co-localization of RECQL4 and P53 to mitochondria, improved *de novo* mtDNA replication and less sensitivity in DNA damage. Deletion mutant of the RECQL4 MTS failed to complement these functions, highlighting the key role of RECQL4 in mitochondrial function (De et al., 2012). Recently, it was also shown that both RECQL4 and P53 regulate the interaction of mitochondrial DNA polymerase γ (PolyA) with mtDNA and thus ensuring mtDNA integrity (Gupta et al., 2014). Apart from the role in mtDNA replication, RECQL4 deficiency cells reduce the mtDNA copy number while overexpression of RECQL4 in HEK293 cells can elevate mtDNA copy number (Chi et al., 2012).

### Nuclear Proteins Modulating ETC Function

#### Signal Transducer and Activator of Transcription 3 (STAT3)

The signal transducer and activator of transcription (STAT) family of proteins are transcription factors involved in a variety of cellular functions including apoptosis, immune responses, tumorigenesis, cell proliferation and autophagy. The family consists of seven members (STAT1, 2, 3, 4, 5A, 5B, and 6). STATs conduct signals from a variety of cytokines through their corresponding receptors with the participation of Janus kinases, JAK1-3 and TYK2. These kinases phosphorylate STATs following cytokine signals leading to their homodimerization and translocation to the nucleus where they exert their function by either activating or repressing nuclear genes. STAT3 has a well-documented role in the nucleus. The canonical pathway of STAT3 activation includes phosphorylation on tyrosine 705 by JAK1, JAK2, and TYK2. These kinases are activated by cytokine signaling (IL1, IL6, LIF, and OSM among others). Tyrosine 705 phosphorylation of STAT3 promotes formation of STAT3 homodimers which enter the nucleus stimulating induction of STAT3-responsive genes. These are involved in development, chronic inflammation, acute phase response, tumorigenesis, DNA damage and metabolism (Yu et al., 2009; Barry et al., 2010; Demaria and Poli, 2011). Activation of *STAT3* gene by phospho-STAT3 leads to elevated levels of unphosphorylated STAT3 which persists for many days in the nucleus. Increased levels of unphosphorylated STAT3 (U-STAT3) in the nucleus induces the expression of a subset of genes quite distinct from that of phosphorylated STAT3, with critical roles in tumorigenesis (Yang et al., 2005; Cheon et al., 2011). Apart from its nuclear role, significant body of evidence shows that STAT3 can function in a non-canonical pathway which includes translocation to mitochondria. Different oncogenic and other stress stimuli trigger mitochondrial translocation of STAT3 in a proteinase K-protected compartment in mouse heart and liver mitochondria (Wegrzyn et al., 2009). Mitochondrial STAT3 co-immunoprecipitates with components of complexes I and II of the respiratory chain. Isolated mitochondria from Stat3^-/-^ pro-B cells exhibit defects in complexes I and II activity. These defects could be specifically rescued by expression of an exclusively mitochondrial STAT3 fusion protein that targets the inner mitochondrial membrane. Mutant analysis showed that Tyr705, DNA binding domain and dimerization of STAT3 is dispensable for its role in ETC regulation, while Ser727 was required. Thus, mitochondrial STAT3 exerts its function in a transcription-independent manner (Wegrzyn et al., 2009). Increased activity of STAT3 correlates with various types of human tumors, while its repression can inhibit tumor progression (Inghirami et al., 2005). Interestingly, upon Ras-dependent malignant transformation, STAT3 was found in mitochondria where it facilitated oncogenic transformation through activation of Complexes II and V. Phosphorylation of Ser727 is required for the oncogenic effect (Gough et al., 2009). Mitochondrial STAT3 has been reported in a variety of cell types and tissues leading to a wide range of cellular outcomes including tumor growth, cell death/survival, and immune regulation. These are achieved through its ability to modulate ETC function, attenuate ROS levels, regulate calcium levels and promote mitochondrial membrane potential and ATP production independently of its role in transcriptional activity (Yang and Rincon, 2016).

### Nuclear Proteins Modulating Cell Death

#### P53

P53 protein is often referred to as “the guardian of the genome” due to its roles in DNA damage repair. As a transcription factor, alongside its DNA repair role, nuclear p53 can regulate diverse cellular functions such as apoptosis, cell cycle, metabolism and autophagy. Several autophagy-related genes have been found among the p53 transcriptional targets. These genes can be specifically induced upon DNA damage both in mouse and human cells and act toward tumor suppression and apoptosis induction in an-undefined manner (Kenzelmann Broz et al., 2013). Conversely, basal levels of cytoplasmic p53 has been shown to inhibit autophagy through inhibition of AMPK and activation of mammalian Target of Rapamycin (mTOR) in (Green and Kroemer, 2009). Apart from its nuclear and cytoplasmic functions, p53 exhibits critical functions upon co-localization with mitochondria (Park et al., 2016). P53 can physically target mitochondria upon pro-apoptotic stimuli where it mediates apoptotic cell death signaling (Marchenko et al., 2000). Mitochondrial accumulation of p53 upon cell death signals requires its mono-ubiquitinylation by the E3 ubiquitin ligase (Marchenko et al., 2007). Numerous pro-apoptotic signals trigger mitochondrial p53 translocation including, cytotoxic agents (Camptothecin), ultraviolet irradiation and hypoxia. On the outer mitochondrial membrane p53 interacts pro-apoptotic and anti-apoptotic members of the Bcl-2 family of cell death regulators, including BAD, BAK, BCL2, and BCLXL, enabling oligomerization of pro-apoptotic members and subsequent cytochrome c release (Mihara et al., 2003; Leu et al., 2004; Jiang et al., 2006). Recently, it was shown that upon oxidative stress p53 translocates to the mitochondrial matrix where it activates the opening of mitochondrial permeability transition pore (MPTP) leading to necrosis (Vaseva et al., 2012). This response is exerted by physical interaction of p53 with Cyclophilin D. Under hypoxic conditions, p53 translocates to mitochondria through its interaction with the mitochondrial chaperone Tid1, also known as mitochondrial Hsp40 (mtHsp40; Ahn et al., 2010). Tid1 depletion attenuated p53 mitochondrial localization and apoptosis induction upon genotoxic or hypoxic signals (Trinh et al., 2010). Artificially matrix-targeted p53 sensitized HepG2 cells to oxidative stress, decreased their respiratory capacity and facilitated cell death (Koczor et al., 2013).

Apart from its role in cell death mitochondrial p53 has been assigned several other functions mainly within the mitochondrial matrix. Indicatively, upon oxidative stress, mitochondrial translocated p53 interacts with manganese superoxide dismutase (MnSOD) reducing its scavenging capacity (Zhao et al., 2005). Furthermore, matrix targeted p53 binds to both mtDNA and mtDNA polymerase gamma (POLG) to promote mtDNA stability and replication capacity of POLG (Achanta et al., 2005), mitochondrial base excision repair (mtBER) (de Souza-Pinto et al., 2004) and accuracy of mtDNA synthesis (Bakhanashvili et al., 2008).

#### Interferon Regulated Factor 3 (IRF3)

RNA virus infection activates the latent transcription factor IRF-3 (Interferon Regulated Factor 3) causing its nuclear translocation and the induction of many antiviral genes. Upon virus infection, a transcriptionally inactive mutant of IRF3 targets mitochondria and promotes apoptosis by directly interacting with pro-apoptotic BAX. The IRF3/BAX interaction, which is mediated by the BH3-like domain of IRF3, recruites BAX onto mitochondria upon viral-RNA infection (Chattopadhyay et al., 2010).

### Nuclear Proteins with Undefined Roles in Mitochondria

#### Signal Transducer and Activator of Transcription 1 (STAT1)

Phosphorylated STAT1 has a well-documented tumor suppressor function in mammalian cells (Kim and Lee, 2007). Nevertheless, nuclear accumulation of unphosphorylated STAT1 (U-STAT1) can also drive the constitutive expression of some genes (Chatterjee-Kishore et al., 2000). In contrast, to the pro-apoptotic function of phosphorylated STAT1, there is convincing evidence that U-STAT1 has anti-apoptotic function and confers resistance to DNA damage (Cheon et al., 2011; Zimmerman et al., 2012; Meissl et al., 2015). Interestingly, STAT1 was shown to localize to mitochondria (Boengler et al., 2010). Specifically, STAT1 resides under physiological conditions in the mitochondrial outer membrane and co-immunoprecipitates with LC3b suggesting a role in mitophagy (Bourke et al., 2013). Further analysis is needed to explore the actual role of the mitochondrial STAT1.

#### Hypoxia-Inducible Factor 1 (HIF-1α)

HIF-1 (hypoxia-inducible factor 1) is a dimeric transcription factor that consists of an O_2_-regulated subunit, Hif-1α, and a constitutively expressed subunit, Hif-1β. HIF-1 is a key regulator of metabolic adaptations based on oxygen availability, conserved in all metazoans species. HIF-1α is constitutively degraded in normoxia in a proteasome-dependent manner. Upon hypoxia HIF-1α is stabilized in the cytoplasm and targets the nucleus where it induces a transcriptional program which confers adaptation to hypoxia. It has been reported that upon hypoxia endogenous HIF-1α targets mitochondria, along with the nucleus in a cell line of human colon carcinoma (Briston et al., 2011). A small fraction of stabilized HIF-1α in normoxia (by proteasomal or prolyl hydroxylase inhibitors) is still able to translocate to mitochondria. This was an interesting observation suggesting a pathway of direct mitochondrial adaptation to changes in oxygen homeostasis. Nevertheless, more work is required to validate whether this is a general phenomenon and elucidate the function of the mitochondrial HIF-1α pool.

## Mitochondrial Proteins Targeting the Nucleus

Several mitochondrial proteins exhibit nuclear localization upon cellular and environmental stimuli. These include transcription factors, enzymes, and pro-apoptotic factors. Their nuclear contribution is mediated by transcriptional or non-transcriptional events (**Table 2**; **Figure 1**). In the following section, we will discuss their dual function in the two compartments and, when available, we will highlight information on the shuttling mechanism.

**Table 2:** Mitochondrial proteins with defined roles in the nucleus.

Protein	Species	Nuclear function	Stimulus driving nuclear localization	Reference
TFAM	Mouse, Rat, Human	Transcription of nuclear genes Promotes cytoprotection against chemotherapeutic drugs	Steady-state conditions	Pastukh et al., 2007; Lee et al., 2014; Tang, 2015
ATFS-1	*C. elegans*	Transcription of genes associated with OXPHOS, glycolysis, mitochondrial chaperons and proteases (UPR^mt^)	Mitochondrial dysfunction Oxidative stress	Jovaisaite and Auwerx, 2015; Tang, 2015
CLK-1/COG-7	*C.elegans*, Human	Promotes the expression of genes associated with oxidative stress response	Mitochondrial dysfunction Increased ROS production	Monaghan et al., 2015; Tang, 2015
Fumarase/FH	Yeast, Human	Production of fumaric acid at the site of DSBs Initiation of the DNA repair machinery Promotes DNA repair and cell survival	Ionizing radiation, Hydroxyurea	Yogev et al., 2010; Jiang et al., 2015; Tang, 2015
PDC	Human	Production of nuclear CoA for histone acetylation	Mitochondrial dysfunction Growth signals (EGF and serum)	Sutendra et al., 2014; Tang, 2015
Nfs1	Yeast	ND	Steady-state conditions	Nakai et al., 2001
MNRR1	Human	Transcription of COX4I2 and its own Affects COX subunits biogenesis and activity	Oxidative or hypoxic stress	Aras et al., 2015
AIF	Yeast Human	Induces DNA fragmentation and chromatin condensation Induces cell death Induces autophagy	Ionizing radiation Anticancer agent FK228	Ye et al., 2002; Watanabe et al., 2009; Sun et al., 2016
HIGD1A	Human	ND	Hypoxic stress and DNA damage	Ameri et al., 2013, 2015

*ND, Not defined.*

### Mitochondrial Proteins with Transcriptional Activity in the Nucleus

#### Transcription Factor A (TFAM)

The mitochondrial transcription factor A (TFAM) is a nuclear encoded protein with mandatory mitochondrial targeting, essential for both transcription and replication of mitochondrial DNA (Ngo et al., 2014). Recently, it has been documented that TFAM was also located in the nuclei and anchored to the chromatin. This dual localization may arise due to harboring two nuclear localization signal (NLS) within the two high mobility group (HMG) domains of TFAM. It has been suggested that TFAM regulates both nuclear and mitochondrial transcription thus promoting cancer cell growth through p21-dependent G1phase cell cycle progression. Furthermore, TFAM confers cytoprotection against genotoxic chemotherapeutic drugs like cisplatin, camptothecin and etoposide (Pastukh et al., 2007; Han et al., 2011). Nuclear localization of TFAM was also observed in hippocampal neuronal cells, where binding of nuclear TFAM to the *tfam* promoter results in the suppression of its own expression in a NRF1-dependent manner. Taken together, these findings suggest that TFAM can regulate gene expression distinctly in each compartment. Mitochondrial TFAM enhances mitochondrial transcription whilst nuclear TFAM suppresses nuclear transcription (Lee et al., 2014).

#### Activating Transcription Factor 1 Associated with Stress (ATFS-1)

In *C. elegans*, the activating transcription factor 1 associated with stress (ATFS-1) is initially localized to mitochondria. Under normal condition, mitochondrial ATFS-1 is constitutively degraded by the Lon protease (Nargund et al., 2012). ATFS-1 amino acid sequence harbors both NLS and MTS allowing the dual localization of the protein. Upon disruption of mitochondrial protein homeostasis or oxidative stress, mitochondrial import rate of ATFS-1 is decreased and a proportion of ATFS-1 remains in the cytosol until its nuclearization. Upon nuclearization, ATFS-1 orchestrates the expression of various genes associated with oxidative phosphorylation, glycolysis, mitochondrial chaperons and proteases. This mitochondria-specific response represents the mitochondrial unfolded protein response (UPR^mt^). Lately, it was shown that a pool of ATFS-1 remains within mitochondria even upon mitochondrial dysfunction. Mitochondrial ATFS-1 binds to the non-coding region of mtDNA and limits the accumulation of mtDNA-encoded transcripts. Similarly, nuclear ATFS-1 reduces expression of specific OXPHOS genes although it induces expression of chaperones, proteases and OXPHOS assembly factors suggesting a role as transcriptional suppressor. Nevertheless, both nuclear and mitochondrial ATFS-1 promote OXPHOS complex assembly and oxygen consumption upon mitochondrial dysfunction, as mutants of either nuclear or mitochondrial or both ATFS-1 forms display defects in OXPHOS assembly and respiration capacity (Nargund et al., 2015). Thus, ATFS-1 is a critical sensor of mitochondrial homeostasis, coordinating both nuclear and mitochondrial genome, although its exact mechanism of action is not clear yet. Similar mitochondrial-specific responses have been also documented in higher eukaryotes, albeit less understood (Jovaisaite and Auwerx, 2015). Recently it was shown that expression of the mammalian ATF5 in *atfs-1* mutant worms was able to activate HSP-60, a key chaperone involved in UPR^mt^ upon mitochondrial stress, rescuing *atfs-1* depletion. Toward this direction, ATF5 deficient HEK293T cells failed to induce the transcription of several mitochondrial chaperons upon paraquat treatment, suggesting a conserved role of ATF5 in UPR^mt^ in mammalian cells similar to ATFS-1 in *C. elegans*. Interestingly, ATF5 is also localized in both mitochondria and nuclei (Fiorese et al., 2016).

#### Clock 1 (CLK-1)

Clock 1 (CLK-1) and its human homolog Coenzyme Q7 Homolog (COQ7), is a mitochondrial enzyme involved in the biosynthesis of ubiquinone which in turn is required for proper ETC function (Rea, 2001; Lu et al., 2013). Both CLK-1and COQ7 expression result in a single translational product which harbors a MTS while only COQ7 contains a defined NTS. Nuclear localization of CLK-1/COQ7 is augmented in response to mitochondrial ROS production (Tang, 2015). Recently, a novel nuclear role of CLK-1/COQ7 has been documented (Monaghan et al., 2015). Using a *clk-1* mutant with impaired MTS which allows for nuclear accumulation of CLK-1 under physiological conditions, it was shown that nuclear CLK-1/COQ7 regulates the induction of oxidative stress response genes and suppression UPR^mt^ genes in an undefined manner (Monaghan et al., 2015; Tang, 2015). Interestingly, the nuclear fraction of COQ7 binds to chromatin and thus may regulate the aforementioned genes transcriptionally (Monaghan et al., 2015). When CLK-1/COQ7 is restricted in the nucleus, ubiquinone biosynthesis is inhibited, inferring that CLK-1/COQ7 perform different functions in the two compartments. Taken together these data support a role for CLK-1/COQ7 as a barometer of oxidative stress within mitochondria, which upon stress translocates to the nucleus triggering the adaptive oxidative stress response, safeguarding mitochondrial homeostasis.

#### Mitochondrial Nuclear Retrograde Regulator 1 (MNRR1)

Mitochondrial nuclear retrograde regulator 1 (MNRR1) is a regulator of cytochrome c oxidase (COX) activity through direct interaction. Under physiological conditions, MNRR1 is transported into mitochondria, where it is predominantly located, through the mitochondrial intermembrane space import and assembly 40 (MIA40) system (Cavallaro, 2010). In response to stress such as low oxygenation, MNRR1 is recruited to the nucleus. Nuclear MNRR1 regulates the transcription of COX4I2 and subsequently the activity of COX. Furthermore, nuclear localization of MNRR1 can enhance its own transcription through a positive feedback loop (Aras et al., 2015).

#### Apoptosis Inducing Factor (AIF)

Apoptosis inducing factor (AIF) is a mitochondrial flavoprotein with pro-apoptotic functions conserved from yeast to mammals. The mitochondrial role of AIF is controversial. It could participate in cellular bioenergetics through its NADH oxidase activity as has been shown *in vitro* (Miramar et al., 2001). It was also suggested to be required for Complex I assembly (Vahsen et al., 2004). More recently, it has been shown that AIF is physically interacting with the mitochondrial coiled-coil-helix-coiled-coil-helix domain containing 4 (CHCHD4), the ortholog of yeast MIA40. Human CHCHD4, similar to MIA40, is involved in the import and oxidative folding of intermembrane space mitochondrial proteins. Due to its capacity to regulate CHCHD4 mitochondrial import, AIF has been proposed to have a wider impact on mitochondrial homeostasis than previously appreciated (Hangen et al., 2015; Modjtahedi and Kroemer, 2016). When cells undergo apoptosis, AIF is released from mitochondria and is accumulated to the nucleus. Nuclear AIF is accompanied with extensive DNA fragmentation and chromatid condensation leading to caspase-independent apoptosis (Ye et al., 2002). Recently, it has been shown that nuclear localization of AIF regulates ionizing radiation (IR) induced cell death (Sun et al., 2016). Previous studies have also associated AIF with autophagy. Particularly, induction of autophagy by supplementation with an anticancer agent (FK228) requires the translocation of AIF to the nucleus (Watanabe et al., 2009). Whilst, the exact mechanism underlying AIF translocation to the nucleus remains unknown, it was suggested that poly (ADP-ribose) polymerase 1 (PARP-1) and human hHR23A, two enzymes with DNA repair activity, may be involved (Yu et al., 2002; Sudhakar and Chow, 2014).

### Mitochondrial Protein with Non-transcriptional Activity in the Nucleus

#### Fumarase (FH)

The enzyme fumarase (FH) is conserved among species and represents a well-known paradigm for dual targeted proteins (Yogev et al., 2010, 2011). Fumarase participates in the tricarboxylic acid cycle (TCA) by catalyzing the reversible conversion of fumarate to malate. Although dual localization and function of FH is conserved among species, the underlying mechanisms are distinct. In yeast, nuclear encoded FH, results in only a single translation product which is synthesized as pro-protein in the cytosol and targeted to mitochondria (Yogev et al., 2010). Under normal conditions, FH pro-proteins harbor a MTS which is cleaved by a mitochondrial processing peptidase (MPP) upon translocation of FH into the mitochondrial matrix (Burak et al., 2013). It was proposed that after the cleavage of MTS, a proportion of FH returns to the cytosol through the reverse translocation mechanism (Yogev and Pines, 2011).

Cytosolic FH can move to the nucleus in response to DNA damage. In an attempt to understand the exact role of FH in both compartments, a yeast strain lacking nuclear FH gene was generated with a copy of FH inserted into its mitochondrial genome. Thus, FH was absent from the cytosol and exclusively present in mitochondria where it preserved TCA function. It has been shown that yeast cells lacking of cytosolic FH exhibit increased sensitivity to DNA damage induced by IR)and hydroxyurea (HU). Exposure to IR and HU cause DSBs and inhibition of DNA synthesis, respectively. When cytosolic FH is present and mitochondrial enzymatic activity is disturbed, yeast cells are again sensitive to DNA damage, suggesting that both cytosolic and mitochondrial activity of FH is required for resistance to DNA damage. However, nuclear FH does not act as a DNA repair protein (Yogev et al., 2010). More recently, it has been documented that nuclear FH can be phosphorylated at Thr236 upon ionizing radiation by the DNA-dependent protein kinase (DNA-PK) holoenzyme. Nuclear accumulation of phosphorylated FH binds to H2A.Z histone variant at the site of DSBs and allows the production of fumarate locally. The phosphorylated FH at the DSBs can inhibit the lysine demethylase 2B (KDM2B) and subsequently control the dimethylation of histone H3 (H3K36me2) at Lys 36. Consecutively, DNA-PK is enriched at DSBs regions promoting non-homologous end-joining (NHEJ) DNA repair and cell survival (Jiang et al., 2015).

#### Pyruvate Dehydrogenase Complex (PDC)

Pyruvate dehydrogenase complex (PDC) is an essential mitochondrial protein complex consisting of three enzymes, the pyruvate dehydrogenase-E1, dihydrolipoamide transacetylase-E2, and dihydrolipoamide dehydrogenase-E3 (Patel et al., 2014). PDC is required for coordination of cytosolic glycolysis and TCA function through converting pyruvate to acetyl-coenzyme A (CoA). In human, it was recently shown that all three subunits of PDC can shuttle from mitochondria to nucleus in a cell cycle-dependent manner. The nuclear pool of PDC can be increased in response to mitochondrial dysfunction, and growth signals, including epidermal growth factor and serum. Similar to its mitochondrial function, nuclear PDC can produce CoA from pyruvate which in turn can be used for histone acetylation (Sutendra et al., 2014; Tang, 2015).

#### NFS1 Cysteine Desulfurase (NFS1)

In *S. cerevisiae*, mitochondrial NFS1 Cysteine Desulfurase (Nfs1) has a cysteine desulfurase activity which must be tightly regulated to preserve and improve mitochondrial metabolism. Nfs1 is a nuclear encoded gene, resulting in a single translational product which is distributed in both mitochondria and nucleus. Nfs1 contains both a MTS and NTS which are required for its dual localization (Yogev and Pines, 2011; Eisenberg-Bord and Schuldiner, 2016). During mitochondrial import, MPP cleaves the MTS of Nfs1 while another processing enzyme, Icp55, further cleaves three amino acids from the N-terminus. Surprisingly, Icp55 is also dually localized to mitochondria and nucleus (Naamati et al., 2009). However, the exact mechanism of Nfs1 and Icp55 targeting to both compartments remains elusive. In the case of Nfs1 nuclear localization, it is suggested that the reverse translocation mechanism is involved (Eisenberg-Bord and Schuldiner, 2016).

#### Hypoxia-Inducible Gene Domain 1A (HIGDA1)

Hypoxia-inducible gene domain 1A (HIGDA1) is a mito chondrial protein interacting with complex IV in yeast, and with complex III in humans (Ameri et al., 2015). Under normal conditions, basal levels HIF-1a regulate expression of mitochondria-localized HIGDA1. These findings imply a role of HIGDA1 in mitochondrial respiration and ROS production. Alterations in metabolic needs as a result of DNA damage and hypoxia can drive nuclear localization of HIGDA1 (Ameri et al., 2013). However, the nuclear function of HIGDA1 as well as the mechanism driving its nuclear localization remains elusive.

## Bi-Organellar Protein Distribution in Aging

Mitochondria are metabolic hubs regulating cellular energy homeostasis, ROS production and stress adaptation, among others. All these key cellular pathways have been causatively liked to the aging process. It is, therefore, widely accepted that mitochondria have decisive roles in aging. Given their pronounced contribution to aging, it is conceivable that mechanisms regulating their ability to adapt to cellular and environmental stimuli, may all have critical implications to the aging process. In the following section we will focus on the reported roles of differentially distributed nuclear or mitochondrial proteins in the context of aging.

### Mitochondrial Pool of Nuclear Transcription Factors Implicated in Aging

Nuclear transcription factors that translocate to mitochondria are master regulators of cellular homeostasis with numerous reports on their role on organismal aging. However, their mitochondrial functions have not been directly linked to aging up to now. Nevertheless, several links to aging related diseases and cellular senescence have been made. Examples are described below.

cAMP response element-binding protein’s role in neuronal plasticity and cognitive function is highly studied. It is known that its activity is reduced in the aged brain and upon neurodegeneration (Sakamoto et al., 2011; Paramanik and Thakur, 2013). The role of mitochondrial CREB in aging is not studied though. However, deregulation of CREB’s mitochondrial targeting is implicated in Huntington Disease (HD). Specifically, a dominant negative mutant of mitochondrial CREB increases vulnerability to 3-nitropropionic acid (3-NP), a mitochondrial toxin that leads to phenotypes close to HD. Mutant huntingtin (htt) can bind CREB, probably sequestering it away from its mitochondrial targets (Lee et al., 2005). Latest findings showed that mutant *htt* blocks mitochondrial protein import by binding to TIM23 import machinery in mouse brain mitochondria, thus causing a more broad effect of mitochondrial biology (Yano et al., 2014).

Myocyte enhancer factor-2D’s role in neuroprotection is well-documented. Although a direct link with aging has not been established yet, mitochondrial MEF2D is implicated in Parkinsons Disease (PD). Mice treated with neurotoxin MPTP (1-methyl-4-phenyl-1,2,3,6-tetrahydropyridine), a precursor molecule of MPP+, exhibit typical pathology of PD (Liberatore et al., 1999). It was reported that MPP+ treatment of mice caused reduced MEF2D and ND6 (MEF2D’s mitochondrial target) protein levels in the brain. Lentivirus-driven expression of an exclusively mitochondrial form of a dominant negative MEF2D mutant (MED2Ddn) significantly accelerated tyrosine hydroxylase (TH) (required for dopamine biosynthesis) signal loss in mouse brain *in vivo*, whilst overexpression of mitochondrial ND6 partially attenuated MPTP-induced TH signal loss (She et al., 2011). Finally, the levels of mitochondrial MEF2D are specifically reduced compared to its nuclear levels in post-mortem brain samples of PD patients, reflecting the observed reduction of ND6 protein levels. Together, these data provide evidence that deregulation of mitochondrial MEF2D and its transcriptional target ND6 could be implicated in the pathogenesis of PD (She et al., 2011).

It has been proposed that accelerated mutation rate in nuclear or mtDNA could be causatively linked to the aging process. A mutant mouse strain expressing an error-prone polymerase gamma (POLG1) accumulates random mutations in its mitochondrial genome and exhibits progeria-like characteristics (Trifunovic et al., 2004). Recently, it was shown that endurance exercise ameliorates the mutational burden and increases lifespan of these mutator mice (Safdar et al., 2015). Moreover, it was proposed that mitochondrial p53, mediates a POLG1-independent mtDNA repair mechanism in response to endurance exercise, underlying a possible role of mitochondrial p53 in regulation of murine lifespan.

Telomere maintenance has a well-documented role in the aging process (Tomas-Loba et al., 2008; Jaskelioff et al., 2011). Although TERT is dowregulated in most non-dividing mammalian tissues, it was shown that overexpression of TERT in epithelial tissue in cancer resistant mice leads to increased median lifespan (Tomas-Loba et al., 2008). Moreover, restoration of telomerase function in a telomerase deficient mouse model with severe tissue degeneration, could reverse age-related deterioration (Jaskelioff et al., 2011). In humans, short telomeres associate with increased risk of mortality (Cawthon et al., 2003). The contribution of mitochondrial TERT to aging is still under investigation. The fact that mitochondrial TERT is important for telomere shortening points to the notion that it could directly affect cellular senescence through its role in telomere dysfunction. It is known that normal fibroblasts expressing the mutant hTERT which fails to exit the nucleus, undergo premature senescence, arrested at the G1/S transition (Kovalenko et al., 2010). However, the cause of senescence, as well as the contribution of the mitochondrion-specific defects to aging remain elusive.

### Nuclear Pool of Mitochondrial Proteins Implicated in Aging

Mitochondrial dysfunction has been correlated with a variety of pathologies, many of which are age-related. Paradoxically, studies in invertebrates revealed a beneficial effect of mild mitochondrial dysfunction in aging and age-related disease models. Evolutionary conservation of this paradox exists in mammals (Liu et al., 2005).

Both *C. elegans* mutants of *clk-*1 and COG7 heterozygous knockout mice display disrupted ubiquinone biosynthesis and live longer than wt populations (Liu et al., 2005). Previously, it was shown that *clk-1* mutation can induce UPR^mt^. Loss of both CLK-1 and UBL-5, a key regulator of UPR^mt^ was able to suppress the longevity phenotype of *clk-1* mutants suggesting a causal role of UPR^mt^ in lifespan extension (Durieux et al., 2011). Interestingly, expression of an exclusively nuclear form of CLK-1 reverses the lifespan extension observed in *clk-1-*null worms. It was shown that nuclear CLK-1 suppress several UPR^mt^ genes without affecting the defects in ubiquinone biosynthesis, implying that it reverses longevity of the mitochondrial mutant by inhibiting UPR^mt^ (Monaghan et al., 2015).

ATFS-1 was shown to translocate to nucleus upon mitochondrial oxidative or proteotoxic stress (Nargund et al., 2012; Houtkooper et al., 2013). Nuclear ATFS-1 activates UPR^mt^ which includes several mitochondrial proteins involved in mitochondrial protein homeostasis, among others (Nargund et al., 2015). This adaptive response has been linked to the aging process (Schulz and Haynes, 2015). ATFS-1 deficiency inhibits the lifespan promoting effects of mild mitochondrial dysfunction. UPR^mt^ can be activated in a cell autonomous and non-autonomous manner. Specifically, mild reduction of mitochondrial function in neurons is able to activate UPR^mt^ in the intestine of nematodes. The latter suggests that a neuronal cue signals to the intestine in order to activate UPR^mt^ (Durieux et al., 2011). This cue is currently unknown.

The causative association of UPR^mt^ to lifespan extension has recently been challenged. Gain-of-function *atfs-1* mutants do not have extended lifespans, and UPR^mt^ activation in short lived mutants of Complex II of the respiratory chain, was not sufficient to extend lifespan of nematodes (Bennett et al., 2014). Conclusively, further studies are needed for the elucidation of the actual role of nuclear ATFS-1 and UPR^mt^ in aging.

## Concluding Remarks

Many nuclear and mitochondrial proteins with bi-organellar distribution have been identified so far, however, our understanding of their roles in the heterologous compartment is limited. Identification of additional players participating in this type of direct bi-organellar communication will be the challenge of future studies. Intriguing implications of mitonuclear coordination with the aging process as well as with age-related diseases are expected to emerge. These advances will expand our understanding and our ability to design and implement new strategies toward holistic treatments promoting quality of life in old age.

## Author Contributions

EL and IG wrote the manuscript. NT reviewed and edited the manuscript.

## Conflict of Interest Statement

The authors declare that the research was conducted in the absence of any commercial or financial relationships that could be construed as a potential conflict of interest.
